# PSMA-PET in the early stages of prostate cancer

**DOI:** 10.1590/0100-3984.2023.56.4e2-en

**Published:** 2023

**Authors:** Felipe de Galiza Barbosa

**Affiliations:** 1 Attending Radiologist in the Department of Radiology and Nuclear Medicine of the Hospital Sírio-Libanês and Americas Group, São Paulo, SP, Brazil. Email: felipegaliza@gmail.com.

In the relentless quest to improve the management of prostate cancer, great advances have been made and improved upon in the diagnostic and therapeutic fields, especially in the last decade. After the role of magnetic resonance imaging (MRI) in locoregional evaluation had been consolidated, the advent of prostate-specific membrane antigen positron emission tomography (PSMA-PET) brought transformations that provided a new perspective on the disease and its management. This advanced imaging method has distinguished itself as a revolutionary tool, redefining the scenario of biochemical recurrence, advanced metastatic disease, and, more recently, the initial staging of patients with prostate cancer^(1)^. Its greater precision in detecting metastatic lesions has increased the accuracy of the diagnosis and guided therapeutic decisions, opening new possibilities for the customization of practice in view of the latest therapeutic advances.

Compared with conventional imaging methods, such as computed tomography (CT), MRI, and bone scintigraphy, PSMA-PET has greater sensitivity and specificity for detecting metastatic disease^(2,3)^. This increase in accuracy comes from the combination of the molecular component of PET and the structural component of CT/MRI, given that PSMA is overexpressed in neoplastic cells within the prostate. That added accuracy is essential to avoid understaging and to allow the therapeutic intervention to be implemented early, thus increasing the likelihood of treatment success and potentially reducing morbidity by averting unnecessary treatments.

In the clinical scenario of the initial staging of prostate cancer, the value of PSMA-PET is more significant in the evaluation of nonprostatic disease such as pelvic lymph node involvement (N), as well as distant, bone and visceral metastases (M1a, M1b, and M1c, respectively), for which conventional methods have major limitations. This assumed advantage of PSMA-PET has become an ally in the therapeutic decision-making process. However, the use of PSMA-PET in the preoperative evaluation of the prostate is a topic that is still under exploration, especially in comparison with the currently available method with higher spatial resolution (MRI) and with histopathological correlation. Therefore, this is a highly relevant, current topic that deserves to be investigated in greater depth in order to improve the diagnostic approach and perhaps to be replicated in scenarios of earlier disease detection.

This editorial congratulates the initiative of Stasiak et al.^(4)^, authors of the article “Preoperative evaluation of prostate cancer by ^68^Ga-PSMA positron emission tomography/computed tomography: comparison with magnetic resonance imaging and with histopathological findings”, published in this issue of Radiologia Brasileira. Their article confirms some premises put forth in the literature^(5)^. The ability of PSMA-PET to detect a lesion within the prostate appears to be similar to that of MRI in the context of the initial staging. I find that to be the most interesting piece of data in the article, because the vast majority of patients were at low or intermediate risk according to the histological grade (International Society of Urological Pathology [ISUP] grade 1-3), which could reduce the detection ability of PET compared with MRI, because the expression of PSMA correlates with the percent Gleason grade pattern 4 in the tumor. That confirms the ability of PSMA-PET to detect cancer and supports the potential extrapolation of its use to an earlier scenario: prostate cancer detection. In addition, it demonstrates the superiority of MRI in detecting extraprostatic extension of a tumor (T3), which is expected given the superior spatial resolution of MRI in comparison with that of CT and PET, because such extension is minimal in the vast majority of cases.

The Stasiak et al.^(4)^ study has some limitations related to its retrospective design. In comparison with conventional methods, PSMA-PET has higher sensitivity and specificity for the initial staging of prostate cancer^(3)^. Despite the limitations, half of the patients did not undergo extended lymphadenectomy (the gold standard for intraoperative confirmation) and there was a low prevalence of positive lymph nodes in the patient sample, which reduces the statistical impact of the comparison. That is evidenced by the fact that the sensitivity of PET was found to be two times greater than that of MRI (44% vs. 22%), although the difference was not statistically significant. Nodal staging is particularly relevant in this clinical scenario, because it has a prognostic value that has been validated in the literature for patients staged as N1 by PSMA-PET and their worse treatment outcomes^(6)^. Therefore, how to use this information in order to reduce morbidity and intensify treatment is a topic currently under debate in the uro-oncology community.

The incorporation of PSMA-PET into the evaluation of the prostate, together with MRI, has progressed to an earlier context: in the detection of prostate cancer in patients with clinical suspicion of the disease. Emmet et al.^(7)^ demonstrated that combining PSMA-PET with MRI increased the sensitivity and the negative predictive value for the detection of clinically significant tumors. That opens the possibility of further exploration of whether PSMA-PET can be used in a variety of potential scenarios, such as in the detection or even active surveillance of clinically non-significant (ISUP grade 1-2) tumors, as well as in guiding biopsies of such tumors, questions to which we should soon have clearer answers.
